# Dysfunction and Post-Traumatic Stress Disorder in Fracture Victims 50 Months after the Sichuan Earthquake

**DOI:** 10.1371/journal.pone.0077535

**Published:** 2013-10-29

**Authors:** Jun Ni, Jan D. Reinhardt, Xia Zhang, Mingyue Xiao, Ling Li, Hong Jin, Xianmin Zeng, Jianan Li

**Affiliations:** 1 Department of Rehabilitation Medicine, The First Affiliated Hospital of Nanjing Medical University, Nanjing, China; 2 Department of Rehabilitation Medicine, The Affiliated Hospital of Nantong University, Nantong, China; 3 Caring For Children Foundation, Hong Kong, China; 4 Institute for Disaster Management and Reconstruction, Sichuan University, Chengdu and Hong Kong Polytechnical University, China; 5 Committee for Rehabilitation Disaster Relief, International Society for Physical and Rehabilitation Medicine, Geneva, Switzerland; 6 Department of Human Functioning Science, Swiss Paraplegic Research, Nottwil, Switzerland; 7 Department of Health Sciences and Health Policy, University of Lucerne, Lucerne, Switzerland; 8 Mianzhu County People’s Hospital, Sichuan, China; 9 Shifang County People’s Hospital, Sichuan, China; University of Pittsburgh, United States of America

## Abstract

**Background:**

This study aimed to evaluate the effectiveness of a rehabilitation intervention on physical dysfunction (PDF) and post-traumatic stress disorder (PTSD) in fracture victims 50 months after the Sichuan earthquake of 2008 and to identify risk factors for PTSD.

**Methods:**

This is a retrospective cohort study. Four hundred and fifty-nine earthquake-related fracture victims from Mianzhu city, Sichuan Province who did not qualify for disability pension participated. Two hundred and forty-five subjects received regular rehabilitation and 214 did not. Muscle strength, joint range of motion (ROM),sensory function, and sit-to-stand balance capacity were evaluated to assess PDF. The PTSD Checklist-Civilian Version (PCL-C) was administered to screen for PTSD. An ordinary least square regression was used to predict PTSD, and a logistic regression was used to predict PDF. In addition a Least Angle Regression (LARS) was carried out for PTSD to study the effects of rehabilitation and PDF at the same time.

**Results:**

Unadjusted and adjusted group differences in physical dysfunction (*p*<0.01) and PTSD prevalence (*p*<0.05) were significant in favor of the rehabilitation group. In addition, being female, average or above family income, having witnessed death and fearfulness were found risk factors for PTSD symptoms 50 months after the earthquake. Both PDF and rehabilitation were selected predictors by LARS demonstrating opposite effects.

**Conclusion:**

PDF and PTSD were significantly reduced by the rehabilitation intervention. Future medical intervention strategies should consider rehabilitation in order to assist survivors in dealing with both physical and psychological effects of natural disaster.

## Introduction

On May 12, 2008, an earthquake of magnitude 8.0 on the Richter Scale struck the densely populated Sichuan Province, in southwestern China. The Sichuan earthquake affected 46 million people resulting in 87,476 deaths, over 350,000 persons injured, leaving many physical and mental health problems in the affected area [1], [2]. Fractures constituted the most common earthquake-related injuries [3]. The need for rehabilitative services increased rapidly after the disaster.

Past research has demonstrated a clear relationship between natural disasters and mental health problems [4], [5], [6], [7]. Studies on the impact of the Sichuan earthquake on the mental health of survivors reported prevalence rates of post-traumatic stress disorder (PTSD) ranging from 9.4% to 45.5% [8], [9].

Comprehensive rehabilitation efforts in the Sichuan earthquake zone, including therapeutic interventions, training and education, as well as vocational and social rehabilitation, were undertaken immediately and concurrently with clinical treatment [3], [10], [11]. Several studies indicated positive effects of rehabilitation on activities of daily living (ADL) and functional health [3], [10], [11], [12], [13], [14], [15]. Furthermore, patients who received rehabilitative training showed improved social integration [10], [16]. Despite these findings, the effectiveness of rehabilitation on recovery from mental health problems remains unclear. Moreover, although the number of devastating earthquakes is very high in China, research on long-term disaster related health problems is limited.

We therefore aimed to examine the effect of rehabilitation on physical dysfunction and PTSD in fracture victims from the 2008 Sichuan earthquake, at 50 months after the disaster.

## Methods

### Design

A cross-sectional survey was conducted in July, 2012, 50 months after the disaster. We employed a retrospective cohort design comparing a group of survivors who underwent regular rehabilitation with a group who did not receive rehabilitation.

### Subjects

We recruited participants from Mianzhu country, an area seriously hit by the earthquake. A total of 459 fracture victims were identified and enrolled. Bodily injury is an important risk factor for PTSD [17], [18], [19]. In order to exclude effects from differing degrees of impairment on PTSD, we selected fracture victims who had well recovered from bodily injury, i.e. at least did not qualify for disability pension. Otherwise, we included patients of all ages (6 to 88 years, mean±std: 53.96±17.76) who had suffered fractures due to the 2008 Sichuan earthquake and had been hospitalized. Based on retrospective information gathered from the victims in personal interviews, subjects were divided into two groups: a rehabilitation group that had reported to have received regular institution-based rehabilitation therapy and a control group that had not.

This research was approved by the Ethics Committee of the Nanjing Medical University and Nantong University. Written informed consent to participate in clinical examinations and surveys was obtained from all participants.

### Measures and Procedures

The research team, which had undertaken three previous large-scale studies, was exclusively comprised of rehabilitation professionals including physiatrists, therapists (physical and occupational), and nurses, all of whom had degrees in rehabilitative medicine and more than 2 years of clinical experience. Members of the team provided both comprehensive evaluation and management of the enrolled subjects. The team performed a critical role in integrating efforts of rehabilitation medical teams from more-developed areas of China and the local healthcare providers [11], [14]. All members had received professional training for accurate evaluation, especially in mental health assessment and communication.

#### Assessment of physical dysfunction

Physical examinations were undertaken by rehabilitation physicians and therapists using muscle strength, joint range of motion (ROM), sensory function and sit-to-stand balance capacity tests. If one or more diagnostic criteria presented a pathology that would require a rehabilitative intervention, we defined it as a physical dysfunction. To avoid interference and bias during assessments, patients and staff performing the examination were blinded, i.e. not advised about the purpose of the examinations. The results were reviewed by other rehabilitation professionals to ensure that no errors were made during data collection.

#### Assessment of earthquake exposures

Four questions were used to evaluate respondents’ earthquake exposures: 1) Was a close family member of you seriously injured or killed? 2) Was your house damaged? 3) Did you feel fear during the earthquake? and 4) Did you directly witness other people die? Four dummy variables were then generated based upon responses to the questions: casualties of family members, house damage, fearfulness during earthquake, and directly witnessing death.

#### Assessment of PTSD

PTSD was assessed with the PTSD Checklist Civilian Version (PCL-C). The PCL-C is a valid and reliable standardized self-reported rating scale for screening PTSD symptoms [20]. It comprises 17 items corresponding to key symptoms of PTSD and is used for any type of traumatic event. In this study, we adopted a version of the PCL-C based on the *Diagnostic and Statistical Manual of Mental Disorders-IV* (DSM-IV, 1994; compiled by the American Research Center for Post-Traumatic Stress Disorder), to identify PSTD-positive survivors. Scores for each question ranged from 1 (not at all) to 5 (extremely). Summing the scores for each of the 17 items provided a total symptom severity score (range = 17–85). Scores between 20–39 are considered mild, 40–59 moderate, 60–79 severe, and scores above 80 extreme PTSD [21].

#### Demographics

Demographic data included information on gender, age, education, income and employment status.

Data on earthquake exposures, PTSD, and demographics were collected in face-to-face interviews. Data were entered by two trained coders into Epidata 3.1. Inconsistencies between the independent coders resulted in verification of the raw data.

### Statistical Analysis

Data were analyzed with SPSS 17.0 and Stata 12.

Descriptive analysis of the data was performed for all variables investigated in the study. Chi-square and t-tests were performed to examine differences between rehabilitation and control group regarding demographics and exposures. A logistic regression model was used to predict the effect of rehabilitation on physical dysfunction. An ordinary least squares (OLS) regression was employed to predict PTSD symptoms. A log transformation of the PTSD scale was applied to meet the assumption of a normal distribution. Both regression models included the following independent variables: gender (reference: female), being married (reference: not married), age, low education (reference: high school education or more), being the main earner of the family (reference: not), low income (reference: income at population average or above), casualties of family members (reference: none), house damage (reference: none), fearfulness during the earthquake (reference: none), directly witnessing death (reference: not), and belonging to the rehabilitation or control group (reference: control). As physical dysfunction and rehabilitation were highly negatively correlated an inclusion of both rehabilitation and dysfunction as predictors into the OLS regression of PTSD seemed inappropriate due to multicollinearity. Therefore, we performed an additional analysis using Least Angle Regression (LARS), i.e. a penalized variable selection algorithm that considers both parsimony and prediction accuracy of the model [22] including physical dysfunction as an additional predictor of PTSD. We expected that both predictors would be selected for optimal model fit and demonstrate opposite effects.

## Results

Four hundred and fifty-nine patients without disability were evaluated, 245 had received rehabilitation and 214 did not participate in rehabilitation. Table 1 provides the sample description and comparison between the rehabilitation group and controls (Table 1). Most victims were females with an average age of around 55, were married, had no or only elementary education, had above average household income, but were not providing the main household income. Control and rehabilitation group did not differ with respect to those demographics. The houses of 445 (96.9%) survivors were damaged. There were significantly more persons in the rehabilitation group whose houses had not been damaged. However, this difference is difficult to interpret due to very small cell counts (the houses of 12 persons in the rehabilitation group and 2 persons in the control group were not damaged).

**Table 1:** Sample description and comparison of study groups.

Variable	Factor	Rehabilitation group (%)	Control group(%)	*X^2^/T*	*p*
**Gender**	Men	90(34.11)	73(36.73)	0.343	0.558
	Women	155(65.89)	141(63.27)		
**Age**	<30	27(11.02)	25(11.68)		
	30–50	71(28.98)	58 (27.10)	0.344	0.952
	50–70	105(42.86)	91(42.52)		
	>70	42(17.14)	40(18.69)		
	Mean±SD	54.25±17.60	53.71±17.93	0.325	0.745
**Marriage**	Married	189(77.14)	167(78.04)	0.180	0.914
	Single	27(11.02)	21(9.81)		
	D or W	29(11.84)	26(12.15)		
**Education**	N	83(33.88)	96(44.86)	6.947	0.139
	E	87(35.51)	67(31.31)		
	M	55(22.45)	38(17.76)		
	H	14(5.71)	11(5.14)		
	C	6(2.45)	2(0.93)		
**Annual family income**	<Average	81(33.06)	57(26.64)	4.102	0.129
	= Average	20(8.16)	12(5.61)		
	>Average	144(58.78)	145(67.76)		
**House damage**	yes	233(95.10)	212(99.07)	6.068	**0.014**
	no	12(4.90)	2(0.93)		
**Bereavement**	yes	96(39.18)	79(36.92)	0.249	0.618
	no	149(60.82)	135(63.08)		
**Witness sb. being killed**	yes	151(61.63)	112(52.34)	4.035	**0.045**
	no	94(38.37)	102(47.66)		
**Fear**	yes	220(89.80)	193(90.19)	0.019	0.089
	no	25(10.20)	21(9.81)		
**Main earner in home**	yes	73(29.80)	58(27.10)	0.406	0.524
	no	172(70.20)	156(72.90)		

Average: Average level, Sichuan provincial average annual family income at the time of the study. D or W: Divorced or Widowed, N: No school education, E: Elementary (Grade 1–6), M: Middle School (Grade 7–9), H: High School (Grade 10–12), C: College or above.

p = probability (significant group differences printed in bold).

Persons in the rehabilitation group had significantly witnessed death more often. Other exposures did not differ between groups (Table 1).

**Figure 1:**
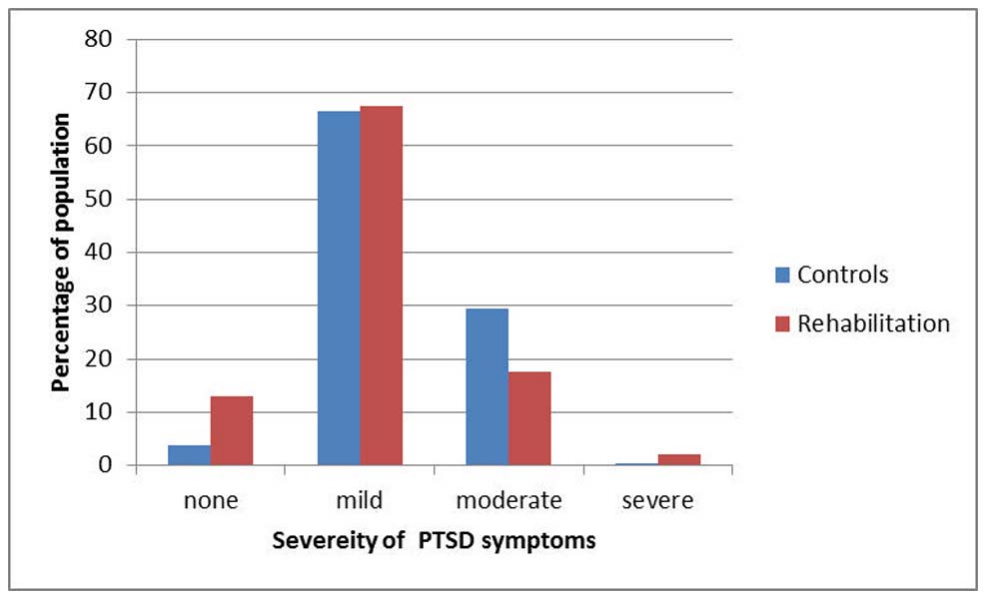
Categorized Post-taumatic Stress Disorder Symptoms accross Study Groups (X^2^ = 20.56, p<0.001).

Unadjusted data show statistically significant differences between the two study groups regarding physical dysfunction (rehabilitation group: 32.24%, control: 67.76%; Chi^2^ = 57.65, p<0.001) as well as PTSD (rehabilitation group: mean = 32.15, SE = 0.7; control group: mean = 34.04, SE = 0.7; t = 1.9, p<0.05), both times in favour of the rehabilitation group (also see Figure 1).

This result is confirmed by the fully adjusted regression models (see Tables 2 and 3), i.e. the likelihood of having been diagnosed with physical dysfunction as well as PTSD symptoms are both significantly decreased in the rehabilitation group. In addition, the likelihood of having physical dysfunction increased with age and for people who had witnessed death. In turn, less PTSD symptoms were reported by men and persons with below average income, while having witnessed people die and fearfullness increased PTSD symptoms.

**Table 2:** Logistic regression of Physical Dysfunction on Selected Predictors.

Predictor	Odds Ratio	SE	z	p
male	0.98	0.27	−0.08	0.935
married	0.96	0.25	−0.18	0.859
age	1.02	0.01	3.19	**0.001**
low education	0.88	0.24	−0.45	0.651
main earner	0.88	0.25	−0.46	0.647
bereavement	0.74	0.16	−1.43	0.154
witnessed death	1.57	0.34	2.07	**0.039**
fearfulness	1.13	0.41	0.35	0.725
low income	0.64	0.15	−1.97	**0.049**
rehabilitation	0.21	0.04	−7.46	**<0.001**
intercept	0.66	0.34	−0.8	0.426

N = 459; Pseudo R-squared = 0.13.

SE = standard Error, z = standardized coefficient, p = probability (significant predictors printed in bold).

**Table 3:** Ordinary Least Squares Regression of Post-traumatic Stress Symptoms (PCLC, log-transformed) on Selected Predictors.

Predictor	Coefficient	SE	t	p
**male**	−0.094	0.036	−2.59	**0.01**
**married**	0.051	0.034	1.49	0.137
**age**	0.001	0.001	1.47	0.143
**low education**	−0.061	0.037	−1.68	0.094
**main earner**	−0.058	0.038	−1.52	0.13
**bereavement**	0.026	0.028	0.9	0.367
**witnessed death**	0.086	0.029	3.02	**0.003**
**fearfullness**	0.252	0.047	5.38	**<0.001**
**low income**	−0.074	0.030	−2.44	**0.015**
**rehabilitation**	−0.070	0.028	−2.54	**0.012**
**intercept**	3.203	0.067	47.98	**<0.001**

N = 459; adjusted R-squared = 0.14.

SE = Standard Error; t = standardized coefficient; p = probability (significant predictors printed in bold).

**Figure 2:**
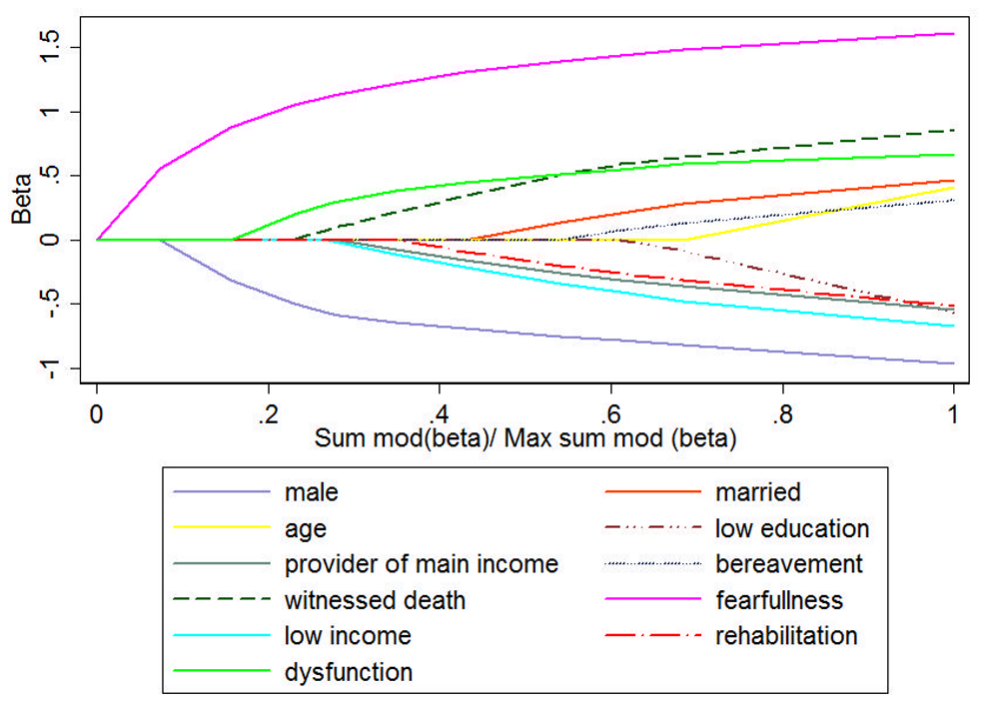
Graphical Depiction of Variable Selection from Least Angle Regression.

The results of the LARS regression of PTSD including physical dysfunction in addition to all above predictors demonstrate that all variables were selected for optimal model fit and that rehabilitation and dysfunction had opposite effects on PTSD, i.e. a positive and a negative effect, respectively (see Table 4 and Figure 2).

**Table 4:** Results from Least Angle Regression of PTSD.

Step	C_p_	R^2^	Action	Coefficient infinal model
**1**	82.17	0.00	Intercept	
**2**	62.76	0.04	+fearfullness	0.2505
**3**	47.46	0.07	+male	−0.0939
**4**	37.74	0.09	+dysfunction	0.0620
**5**	34.37	0.10	+saw people die	0.0807
**6**	34.87	0.11	+low income	−0.0681
**7**	29.47	0.12	+main eaner	−0.0559
**8**	23.93	0.13	+rehab	−0.0482
**9**	18.11	0.15	+married	0.0518
**10**	16.45	0.16	+family member died	−0.0295
**11**	15.51	0.16	+low education	−0.0598
**12**	**12.00**	0.17	+age	0.0011

C_p_ = Estimate of prediction error (minimum printed in bold).

## Discussion

This study was designed to examine the impact of rehabilitation on physical dysfunction and PTSD symptoms in fracture victims of the 2008 Sichuan earthquake. The present study demonstrates that the incidence of physical dysfunction and PTSD at 50 months after the disaster was significantly reduced in survivors who had underwent rehabilitation as compared to survivors who had not while controlling for demographics and earthquake exposure.

While our finding on physical dysfunction confirms previous research showing that patients with fractures who underwent rehabilitative therapies had better functional outcomes and reduced complications, and thus an improved quality of life (QoL) [3], [10], [11], a long-term effect of physical rehabilitation on PTSD had not been reported previously. There are several possible explanations for the latter finding: 1) Rehabilitation improved functional health, and improved functioning may have contributed to better mental health. Previous research has shown that severe physical impairment and chronic pain are important contributing factors to long-term PTSD in the aftermath of a traumatic accident [23], [24], [25]. In a study of war veterans from Afghanistan and Iraq, Jakupcak and colleagues, for instance, found that PTSD symptom severity was significantly associated with poorer health functioning, even after accounting for demographic factors, combat and chemical exposure and health risk behaviors [26]. 2) With a reduction in symptom severity, survivors may have had an improved ability to return to educational, work and family responsibilities. Better social participation in turn may have a direct effect on mental health. 3) The physical rehabilitation programming included physical exercise and instructions for home exercise. Positive influence of exercise on mental health has been documented previously [27], [28], [29]. 4) Unlike some other clinical therapies, rehabilitation is a multidisciplinary intervention strategy that is more interactively concerned with patient psychology. As rehabilitation requires the active participation of patients, physicians and therapists need more often actively communicate with patients to ensure their contribution. Moreover, most physicians and therapists who provided rehabilitation after the 2008 Sichuan earthquake had received some psychological training, particularly in relation to PTSD. All these unique components of rehabilitation may have contributed to improved PTSD outcomes. This explanation is further supported by our finding from the LARS regression on PTSD showing a positive association of dysfunction and PTSD as well as an independent effect of rehabilitation.

Many studies suggest that disasters and other traumatic events have short and long term health consequences,especially as regards the occurrence of functional disabilities as well as psychiatric disorders including PTSD, major depression, and anxiety [30], [31]. It was also found that female gender, older age, bodily injury and bereavement as well as earthquake exposures are important risk factors associated with PTSD among both adolescents [17], [19] and adults [9], [18].

In our study, apart from not having received physical rehabilitation and physical dysfunction, main risk factors for PTSD appeared to be female gender, having witnessed people die, fearfulness, and average or above income, while bereavement and home damage did not play a major role.

Our finding that females are at higher risk for PTSD is consistent with previous research [8], [17], [19], [30]. It further needs to be considered that Mianzhu is a rural setting within which women play non-dominant roles regarding productivity and culture; external locus of control and rather passive maladaptive coping may be a consequence. However, more research is warranted to confirm this hypothesis.

Differential earthquake exposures have been identified as important psychological risk factors before. Similar to our findings, Salcioguo and colleagues showed that PTSD was strongly related to fear during an earthquake in a sample of 769 Turkish survivors who had been relocated to a permanent housing site built in the epicentre region three years after the disaster [32].

Our result on witnessing death as a contributory factor for PTSD symptoms confirms findings from previous studies conducted 15 months [33] and 3 years [34] after the Sichuan earthquake.

In contrast, to previous research we did not find a significant influence of having lost a family member in our study population. As our study was conducted more than 4 years after the disaster, it is likely that our participants had already accepted their losses and were no longer in grief.

Our finding on house damage not being a risk factor is difficult to interpret as solely 14 persons (3%) reported no damage of their residence and we do not have information on the severity of the damage. Moreover, at the time of the study most survivors had moved to new buildings that were better equipped and constructed than those previously damaged.

Eventually, our result regarding higher income being a risk factor is surprising and not consistent with previous studies [8], [18]. A likely explanation for this inconsistent finding is that our study has been conducted in a relatively underdeveloped setting. People with low family income may be very much occupied with meeting needs of daily living and accordingly distracted from psychological problems. Moreover income and education are closely correlated and psychological disorders are less well known to and less accepted in persons with low educational levels [35].

Some possible limitations of our study need to be addressed. There was no baseline data on PTSD of the interviewed participants shortly after the earthquake so that we could not study change or adjust for potential baseline differences between the study groups. However, since demographics and most exposures were similar (even a higher percentage of participants from the rehabilitation group witnessed death) baseline differences in favour of the rehabilitation group seem unlikely. Secondly, we did not address other mental health problems, such as anxiety and depression so that we do not have a complete picture of the mental health of the study population at the moment; an issue to be addressed by future research. Thirdly, we used a screening tool for PTSD and did not confirm these results with more intensive psychiatric examination, particularly due to resource constraints. Finally, several predictors in our models such as income and education were correlated which makes it difficult to demonstrate separate effects. However, additional analysis using penalized regression (LARS) showed that inclusion of all selected predictors resulted in optimal model fit.

In conclusion, this is the first study to examine the association of rehabilitation and PTSD symptoms in Chinese fracture victims after the 2008 Sichuan earthquake. Our study suggests a positive impact of medical rehabilitation on PTSD symptoms. This finding may have important implications for prevention and intervention in post- traumatic mental health problems in survivors of natural disasters.

## References

[1] China SCIOo (2008) The update statistic report of Wenchuan earthquake (in Chinese). http://www.scio.gov.cn/gzdt/ldhd/200809/t222722htm (Accessed 17 Apr 2013).

[2] You C, Chen X, Yao L (2009) How China responded to the May 2008 earthquake during the emergency and rescue period. J Public Health Policy 30: 379–393; discussion 393–374. 10.1057/jphp.2009.30 20029427

[3] Xiao M, Li J, Zhang X, Zhao Z (2011) Factors affecting functional outcome of Sichuan-earthquake survivors with tibial shaft fractures: a follow-up study. J Rehabil Med 43: 515–520. 21533332 10.2340/16501977-0813

[4] Pandya A (2013) A review and retrospective analysis of mental health services provided after the september 11 attacks. Can J Psychiatry 58: 128–134. 23461883 10.1177/070674371305800302

[5] Price M, Davidson TM, Andrews JO, Ruggiero KJ (2013) Access, use and completion of a brief disaster mental health intervention among Hispanics, African-Americans and Whites affected by Hurricane Ike. J Telemed Telecare. 10.1177/1357633X13476230 PMC3676648 23514936

[6] van Griensven F, Chakkraband ML, Thienkrua W, Pengjuntr W, Lopes Cardozo B, et al (2006) Mental health problems among adults in tsunami-affected areas in southern Thailand. JAMA 296: 537–548. 16882960 10.1001/jama.296.5.537

[7] Chou YJ, Huang N, Lee CH, Tsai SL, Tsay JH, et al (2003) Suicides after the 1999 Taiwan earthquake. Int J Epidemiol 32: 1007–1014. 14681266 10.1093/ije/dyg296

[8] Kun P, Chen X, Han S, Gong X, Chen M, et al (2009) Prevalence of post-traumatic stress disorder in Sichuan Province, China after the 2008 Wenchuan earthquake. Public Health 123: 703–707. 19892379 10.1016/j.puhe.2009.09.017

[9] Wang L, Zhang Y, Wang W, Shi Z, Shen J, et al (2009) Symptoms of posttraumatic stress disorder among adult survivors three months after the Sichuan earthquake in China. J Trauma Stress 22: 444–450. 19718756 10.1002/jts.20439

[10] Zhang X, Reinhardt JD, Gosney JE, Li J (2013) The NHV rehabilitation services program improves long-term physical functioning in survivors of the 2008 Sichuan earthquake: a longitudinal quasi experiment. PLoS One 8: e53995. 23308293 10.1371/journal.pone.0053995 PMC3538750

[11] Hu X, Zhang X, Gosney JE, Reinhardt JD, Chen S, et al (2012) Analysis of functional status, quality of life and community integration in earthquake survivors with spinal cord injury at hospital discharge and one-year follow-up in the community. J Rehabil Med 44: 200–205. 22367060 10.2340/16501977-0944

[12] Li Y, Reinhardt JD, Gosney JE, Zhang X, Hu X, et al (2012) Evaluation of functional outcomes of physical rehabilitation and medical complications in spinal cord injury victims of the Sichuan earthquake. J Rehabil Med 44: 534–540. 22674233 10.2340/16501977-1005

[13] Zhang X, Hu XR, Reinhardt JD, Zhu HJ, Gosney JE, et al (2012) Functional outcomes and health-related quality of life in fracture victims 27 months after the Sichuan earthquake. J Rehabil Med 44: 206–209. 22367105 10.2340/16501977-0945

[14] Gosney J, Reinhardt JD, Haig AJ, Li J (2011) Developing post-disaster physical rehabilitation: role of the World Health Organization Liaison Sub-Committee on Rehabilitation Disaster Relief of the International Society of Physical and Rehabilitation Medicine. J Rehabil Med 43: 965–968. 22031340 10.2340/16501977-0890

[15] Castillo RC, MacKenzie EJ, Archer KR, Bosse MJ, Webb LX, et al (2008) Evidence of Beneficial Effect of Physical Therapy After Lower-Extremity Trauma. Archives of Physical Medicine and Rehabilitation 89: 1873–1879. 18929015 10.1016/j.apmr.2008.01.032

[16] Samuelkamaleshkumar S, Radhika S, Cherian B, Elango A, Winrose W, et al (2010) Community reintegration in rehabilitated South Indian persons with spinal cord injury. Arch Phys Med Rehabil 91: 1117–1121. 20599052 10.1016/j.apmr.2010.04.005

[17] Ma X, Liu X, Hu X, Qiu C, Wang Y, et al (2011) Risk indicators for post-traumatic stress disorder in adolescents exposed to the 5.12 Wenchuan earthquake in China. Psychiatry Research 189: 385–391. 21295350 10.1016/j.psychres.2010.12.016

[18] Kun P, Han S, Chen X, Yao L (2009) Prevalence and risk factors for posttraumatic stress disorder: a cross-sectional study among survivors of the Wenchuan 2008 earthquake in China. Depress Anxiety 26: 1134–1140. 19842170 10.1002/da.20612

[19] Fan F, Zhang Y, Yang Y, Mo L, Liu X (2011) Symptoms of posttraumatic stress disorder, depression, and anxiety among adolescents following the 2008 Wenchuan earthquake in China. J Trauma Stress 24: 44–53. 21351164 10.1002/jts.20599

[20] Conybeare D, Behar E, Solomon A, Newman MG, Borkovec TD (2012) The PTSD Checklist-Civilian Version: reliability, validity, and factor structure in a nonclinical sample. J Clin Psychol 68: 699–713. 22517497 10.1002/jclp.21845

[21] Grubaugh AL, Elhai JD, Cusack KJ, Wells C, Frueh BC (2007) Screening for PTSD in public-sector mental health settings: the diagnostic utility of the PTSD checklist. Depress Anxiety 24: 124–129. 16892418 10.1002/da.20226

[22] Bradley Efron TH, Iain Johnstone, Robert Tibshirani (2004) Least angle regression. Ann Statist 32: 407–499.

[23] Barth J, Kopfmann S, Nyberg E, Angenendt J, Frommberger U (2005) Posttraumatic stress disorders and extent of psychosocial impairments five years after a traffic accident. Psychosoc Med 2: Doc09. 19742068 PMC2736498

[24] Blanchard EB, Hickling EJ, Forneris CA, Taylor AE, Buckley TC, et al (1997) Prediction of remission of acute posttraumatic stress disorder in motor vehicle accident victims. J Trauma Stress 10: 215–234. 9136089 10.1023/a:1024826028483

[25] Mayou R, Tyndel S, Bryant B (1997) Long-term outcome of motor vehicle accident injury. Psychosom Med 59: 578–584. 9407575 10.1097/00006842-199711000-00004

[26] Jakupcak M, Luterek J, Hunt S, Conybeare D, McFall M (2008) Posttraumatic stress and its relationship to physical health functioning in a sample of Iraq and Afghanistan War veterans seeking postdeployment VA health care. Journal of Nervous and Mental Disease 196: 425–428. 18477887 10.1097/NMD.0b013e31817108ed

[27] Tordeurs D, Janne P, Appart A, Zdanowicz N, Reynaert C (2011) Effectiveness of physical exercise in psychiatry: A therapeutic approach? Encephale-Revue De Psychiatrie Clinique Biologique Et Therapeutique 37: 345–352. 10.1016/j.encep.2011.02.003 22032277

[28] Rosenbaum S, Nguyen D, Lenehan T, Tiedemann A, van der Ploeg HP, et al (2011) Exercise augmentation compared to usual care for post traumatic stress disorder: a randomised controlled trial (the REAP study: Randomised Exercise Augmentation for PTSD). BMC Psychiatry 11: 115. 21777477 10.1186/1471-244X-11-115 PMC3151207

[29] Schulz KH, Meyer A, Langguth N (2012) [Exercise and psychological well-being]. Bundesgesundheitsblatt Gesundheitsforschung Gesundheitsschutz 55: 55–65. 22286249 10.1007/s00103-011-1387-x

[30] Stratta P, de Cataldo S, Bonanni R, Valenti M, Masedu F, et al (2012) Mental health in L’Aquila after the earthquake. Ann Ist Super Sanita 48: 132–137. 22751555 10.4415/ANN_12_02_05

[31] Yzermans CJ, Donker GA, Kerssens JJ, Dirkzwager AJ, Soeteman RJ, et al (2005) Health problems of victims before and after disaster: a longitudinal study in general practice. Int J Epidemiol 34: 820–826. 15860632 10.1093/ije/dyi096

[32] Salcioglu E, Basoglu M, Livanou M (2007) Post-traumatic stress disorder and comorbid depression among survivors of the 1999 earthquake in Turkey. Disasters 31: 115–129. 17461919 10.1111/j.1467-7717.2007.01000.x

[33] Jia Z, Tian W, Liu W, Cao Y, Yan J, et al (2010) Are the elderly more vulnerable to psychological impact of natural disaster? A population-based survey of adult survivors of the 2008 Sichuan earthquake. BMC Public Health 10: 172. 20353554 10.1186/1471-2458-10-172 PMC2867995

[34] Wen J, Shi YK, Li YP, Yuan P, Wang F (2012) Quality of life, physical diseases, and psychological impairment among survivors 3 years after Wenchuan earthquake: a population based survey. PLoS One 7: e43081. 22937014 10.1371/journal.pone.0043081 PMC3427990

[35] Phillips MR (2013) Can China’s new mental health law substantially reduce the burden of illness attributable to mental disorders? Lancet 381: 1964–1966. 23746885 10.1016/S0140-6736(13)61177-0

